# The Outer Membrane Protein OmpW Enhanced *V. cholerae* Growth in Hypersaline Conditions by Transporting Carnitine

**DOI:** 10.3389/fmicb.2017.02703

**Published:** 2018-01-22

**Authors:** Xiuping Fu, Jingyun Zhang, Tianyi Li, Mei Zhang, Jie Li, Biao Kan

**Affiliations:** ^1^State Key Laboratory of Infectious Disease Prevention and Control, National Institute for Communicable Disease Control and Prevention, Chinese Center for Disease Control and Prevention, Beijing, China; ^2^Collaborative Innovation Center for Diagnosis and Treatment of Infectious Diseases, Hangzhou, China

**Keywords:** *Vibrio cholerae*, salt stress, outer membrane protein, OmpW, osmoadaptation, compatible solute, carnitine

## Abstract

Pathogenic marine bacteria are found in environments and food sources with high salt concentrations, which the bacteria must effectively manage for their survival. Several mechanisms, such as the transport of ions and compatible solutes as well as changes in aerobic and anaerobic respiration, confer salt tolerance to bacteria. In this study, we found that the outer membrane protein OmpW was related to salt stress in *Vibrio cholerae* and that *ompW* gene transcription and expression were up-regulated in cultures containing high NaCl concentrations. Deletion of *ompW* resulted in reduced *V. cholerae* growth in hypersaline culture conditions. Supplements of the compatible solutes betaine, *L*-carnitine, or *L*-lysine enhanced the growth of *V. cholerae* in hypersaline media. Supplements of betaine or *L*-lysine had the same growth enhancement effect on the *ompW*-deletion mutant cultured in hypersaline media, whereas *L*-carnitine supplementation did not restore mutant growth. In addition, the uptake of *L*-carnitine was decreased in the *ompW*-deletion mutant. Our study showed that among the multiplex factors that enhance the hypersaline tolerance of *V. cholerae*, OmpW also plays a role by transporting *L*-carnitine.

## Introduction

Salinity osmotic pressure is an inevitable environmental pressure for all microorganisms, especially marine bacteria. The ability to tolerate salt is important for bacteria to survive and thrive in severe environments. Many bacterial pathogens in the marine environment, such as *Vibrio* and *Shewanella*, are a threat to human health through the seafood supply, and resistance to the high salt levels in food is a prerequisite for the survival and pathogenesis of these food-borne pathogens in humans.

Studies of the osmoregulation of bacteria have shown that most bacteria exclude Na^+^ and take up K^+^ when exposed to high-salt conditions (Roesser and Müller, 2001; Hengge-Aronis, 2002). A high concentration of cytoplasmic K ions can result in suboptimal cytoplasmic conditions for cell growth; thus, bacteria will accumulate compatible solutes to balance the osmotic strength of the cytoplasm (Landfald and Strøm, 1986; Cayley et al., 1992; Verheul et al., 1998; Bourot et al., 2000; Shahjee et al., 2002). The compatible solutes do not interfere with central cell metabolism, even when they accumulate to high concentrations (Brown, 1976). Compatible solutes include two major groups: (i) amino acids and amino acid derivatives, such as lysine, proline, choline, betaine, and carnitine; and (ii) sugars and polyols, such as trehalose, mannitol, and taurine. In addition to their biosynthesis by microorganisms, compatible solutes can accumulate via uptake from the environment.

Bacterial outer membrane proteins play an important role in adaptation to salt stress because of their location: directly contacting the high-salt environment and the channels involved in substance transport. Outer membrane proteins are osmoregulation-sensitive in some bacteria such as *Listeria monocytogenes, Vibrio alginolyticus*, and *V. parahaemolyticus* (Jalajakumari and Manning, 1990; Xu et al., 2004, 2005). OmpW is a member of a major protein family that localizes to the bacterial outer membrane and is involved in the transport of small hydrophobic molecules and iron (Thompson et al., 2002; Hong et al., 2006; Gil et al., 2007). OmpW may confer salt tolerance to *Photobacterium damselae* (Wu et al., 2006), and increased expression of OmpW has been observed in response to high NaCl concentrations in *V. alginolyticus* (Xu et al., 2004) and *V. parahaemolyticus* (Xu et al., 2005), but the exact mechanism of these processes is still unknown.

*V. cholerae* is an important human intestinal pathogen and often survives and thrives in estuaries and high-salt food, which underscores the ability of the bacterium to tolerate high-salt conditions. *V. cholerae* can grow in the presence of 0.5 to 5% NaCl, although low salinity (0.5–2%) conditions are optimal for its growth (Fu et al., 2014), suggesting that *V. cholerae* has a powerful salt-regulation system. We have found that the salt-related genes encoding Na^+^ exclusion, K^+^ uptake, glutamate biosynthesis, and some sigma factors are up-regulated in response to salt stress in *V. cholera* (Fu et al., 2014). The regulator OscR was found to modulate the transcription of genes involved in biofilm matrix production and motility in a salinity-dependent manner (Shikuma and Yildiz, 2009). The uptake of certain compatible solutes, including ectoine, glycine betaine, and proline, can enhance the osmoadaptation ability of *V. cholerae* (Pflughoeft et al., 2003; Kapfhammer et al., 2005). These combined strategies may contribute to the persistence of *V. cholerae* in marine environments and high-salt food. The gene encoding OmpW is also present in *V. cholerae* strains (Nandi et al., 2005) and has been widely used as a specific target for the detection and identification of *V. cholerae* (Nandi et al., 2000), although the biological significance of OmpW in *V. cholerae* is unknown.

In this study, we focused on the possible role of OmpW in osmoadaptation in *V. cholerae*. We found that *ompW* was up-regulated in response to high NaCl concentrations and elevated the salt tolerance of *V. cholerae*. This enhanced salt tolerance was achieved through the transport of carnitine, a compatible solute that may enhance the osmoadaptation of *V. cholerae*.

## Materials and methods

### Bacterial strains and chemical reagents

*V. cholerae* El Tor biotype strain C6706 was used in this study. All experiments involving the live *V. cholerae* such as the bacteria culture and the bacteria inactivation were operated in the BSL-2 Laboratory.

Luria-Bertani (LB) medium was purchased from Oxoid (UK). Na_2_HPO_4_, KH_2_PO_4_, NaCl, NH_4_Cl, MgSO_4_, CalCl_2_, and glucose were purchased from Sangon Biotech (CHN). HPLC-grade acetonitrile (ACN), methanol (MeOH), and formic acid (FA) were purchased from Fisher Scientific (NJ, USA). Water was prepared by a Milli-Q system (Millipore, MA, USA).

### Measurement of transcription and expression of *ompW* under salt stress

#### RNA extraction and qRT-PCR

*V. cholerae* strains were cultivated overnight at 37°C, diluted to an OD_600_ of 1.0 and then used for seed cultures. The seed cultures were diluted 1:100 and then cultivated in triplicate in M9 medium (1.3% NaH_2_PO_4_·7H_2_O, 0.3% K_2_HPO_4_, 0.1% NH_4_Cl, 2 mM MgSO_4_, and 100 μM CaCl_2_) supplemented with 0.5% glucose and 0.5, 2, 4, or 5% NaCl at 37°C and 200 rpm for 1 h. The cultures were harvested, and total RNA was extracted using RNAiso reagent (TaKaRa). Total RNA extraction, qRT-PCR assays, and identification of the internal control gene were performed as in our previous study (Fu et al., 2014). To identify the most stable internal control gene, 6 housekeeping genes were selected and evaluated using geNorm software. M values were calculated according to method mentioned in previous studies (Vandesompele et al., 2002; Zhang Cuicai et al., 2014).

The relative expression of the *ompW* gene was determined using the equation 2-^ΔΔcq^, and the expression level at 0.5% NaCl was used as the baseline value, with the *thyA* gene serving as an internal control. The specific primers used to determine the transcript levels were *ompW*-F (5′- CGC GGG TAT TGC CTC GGT AGT A−3′) and *ompW*-R (5′ -ATC TTA TGT GAA AAT GGC GTA GCA−3′).

#### Western blotting

*V. cholerae* cells were cultivated overnight at 37°C, diluted to an OD_600_ of 1.0 and then used as seed cultures. The seed cultures were diluted 1:100 and then cultivated in triplicate in M9 medium containing 0.5, 2, 4, or 5% NaCl at 37°C and 200 rpm until the early stationary phase (in the M9 media containing 0.5 and 2% NaCl, the cells were incubated for ~12 h to reach the OD_600_ value of 0.8; for media containing 4 and 5% NaCl concentrations, the cells were incubated for ~18 h to reach an OD_600_ value of 0.5). Cells were pelleted by centrifugation at 10,000 g for 5 min at 4°C. Cell pellets were suspended in PBS, and the cell density was adjusted to an OD_600_ of 0.6. Pellets from 6 mL aliquots of the above samples were then suspended in 100 μL of RIPA Lysis Buffer (CWBIO) and incubated for 30 min on ice. The samples were centrifuged at 10,000 g for 5 min at 4°C, the supernatants were collected, and the proteins were quantified with BCA Protein Assay Kit (Thermo). Equal quantities (20 μg) of each sample were mixed separately with loading buffer. Protein samples were separated by SDS-PAGE (12%), then transferred onto PVDF membranes, and analyzed by western blotting using a rabbit anti-OmpW polyclonal antibody (prepared in our laboratory) and an anti-*E. coli* CRP antibody (BioLegend, USA) (CRP expression was used as the internal control). The secondary antibodies that were used were horseradish peroxidase (HRP)-conjugated goat anti-rabbit and goat anti-mouse antibodies. Membranes were visualized using an HRP-DAB substrate coloration assay kit (TIANGEN, China). Images were obtained by scanning the membrane, and the integrated density (IntDen) values of the OmpW and CRP hybridization bands in each lane were calculated with ImageJ software (Schneider et al., 2012). The ratio between the IntDen of the OmpW and CRP bands in each lane (which contained samples grown at different NaCl concentrations) was calculated to estimate the relative OmpW expression.

### Mutant construction and gene complementation

Mutants in which *ompW* was deleted were constructed by homologous recombination using the suicide plasmid pwM91 in *V. cholerae* C6706, as previously described (Xu et al., 2014). The deletion fragment inserted into pwM91 was produced using overlap extension PCR. The two primer pairs pwM91F1/pwM91R1 and pwM91F2/pwM91R2 (Table 1) were used to amplify the upstream and downstream regions, respectively, of the *ompW* gene in C6706 chromosomal DNA.

**Table 1:** Primers used in this study.

**Primers**	**Sequence**	**Restriction site**
pwM91F1	ATAAGAATGCGGCCGCAATCCCTTTACTGGACTCGGTT	*Not*I
pwM91R1	GGAAAACGTCCGCCCTATTTCGAAAATAAA	
pwM91F2	AAATAGGGCGGACGTTTTCCTTTTTTGT	
pwM91R2	CCGCTCGAGATACGGTCTGGCGTGCTGAG	*Xho*I
*ompW*-F	GGAATTCGGCAATGGTATTAACGGCTTC	*EcoR*I
*ompW*-R	CGGGATCCTTAGAACTTATAACCACCCGCGA	*BamH*I

The *ompW* ORF was amplified using the primers *ompW*-F and *ompW*-R, which contained *EcoR*I and *BamH*I restriction sites, respectively (Table 1). The PCR fragments were digested with *BamH*I and *EcoR*I (TaKaRa) and ligated into the plasmid pBR322 (D3050; TaKaRa), which had been digested with the same enzymes. The plasmid was then electroporated into the *ompW*-deletion mutant strains.

### Scanning electron microscopy

The seed cultures were diluted 1:100 and then cultivated in triplicate in M9 medium containing 0.5% NaCl at 37°C and 200 rpm until OD_600_ reached 0.5. The cultures were harvested, then fixed in 4% glutaraldehyde. After dehydration in ethanol, the dried specimens were attached to metal stubs with silver paste and sputter-coated with gold/palladium, thickness 30 nM in a vacuum evaporator. Cells were examined in a scanning electron microscope (30 ESEM; Philips, Eindhoven, The Netherlands).

### Salinity tolerance of the *V. cholerae* strains

The salinity tolerance of the wild-type *V. cholerae* strain C6706, the *ompW* mutant strain C6706-Δ*ompW* and the complementation strain C6706-Δ*ompW/compW* was examined. The *V. cholerae* seed cultures were diluted to a final OD_600_ of 1.0. The seed cultures were then diluted 1:100 into 5 mL of M9 medium supplemented with 0.5% glucose containing different concentrations of NaCl (0.5 and 5%). The cultures were cultivated at 37°C and 200 rpm for 18 h. The bacterial counting was measured every 6 h in triplicate. The OD_600_ of the cultures was measured spectrophotometrically each hour using a TECAN Infinite M200 Pro. For every result, the average OD_600_ values were calculated from a minimum of three independently inoculated growth trials. The relative differences in the mean 18 h OD_600_ values were determined for the wild-type *V. cholera*e strains, the *ompW* mutants and the complementation strains. Two-tailed *t*-tests with *P*-values ≤ 0.05 were considered to be indicative of data with a significant difference.

### Identification of osmoprotectant candidates transported by OmpW

The *V. cholerae* seed cultures were diluted from LB broth overnight cultures to a final OD_600_ of 1.0. The seed cultures were subsequently diluted 1:100 into 5 mL of M9 medium (supplemented with 0.5% glucose) containing 0.5 or 5% NaCl. Seven different osmoprotectants (*L-*carnitine, betaine, *L*-arginine, *L*-proline, *L*-taurine, *L*-trehalose, and *L*-lysine) were added to culture media at a concentration of 5 mM. The osmoprotectants were filter-sterilized using a 0.22 μm filter. The cultures were incubated at 37°C and 200 rpm for 18 h, and their OD_600_ was monitored spectrophotometrically as described previously. The relative differences in the mean 18 h OD_600_ values of the culture media with osmoprotectants and the culture media without osmoprotectants were determined. Statistical significance of the growth differences was determined as described for the salt tolerance studies. The pH of all the above media was adjusted to 7.0.

### Quantitative analysis of carnitine by liquid chromatography multiple reaction monitoring mass spectrometry (LC-MRM-MS)

#### Extraction of carnitine

The wild-type *V. cholerae* C6706 and the *ompW* mutant C6706-Δ*ompW* strain seed cultures were diluted from LB broth overnight cultures to a final OD_600_ of 1.0. The seed cultures were then diluted 1:100 into 200 mL of M9 medium containing 5% NaCl with 6 mM *L-*carnitine (this point was defined as time zero). The cultures were incubated at 37°C and 200 rpm for 10 h. At two time points (0 and 10 h), 50 mL of cultivated bacterial medium was centrifuged at 5,500 g for 10 min at 4°C to obtain the supernatant culture medium as biological triplicates. The supernatant culture medium samples were then transferred into 2 mL EP tubes and then lyophilized completely. Next, each sample was subjected to ultrasound extraction for 15 min in 1 mL of MeOH and then centrifuged at 15,000 g for 5 min at room temperature. The MeOH phases were combined and dried by nitrogen after the ultrasound extraction was repeated three times.

#### LC-MS/MS analysis

A Waters ACQUITY UPLC system was coupled with a Thermo Fisher UltiMate 3000 UHPLC and an Aglient ZORBAX 300SB-C18 column (250 × 4.6 mm, 2.6 μm) and operated at a flow rate of 0.2 mL·min^−1^ for quantitative analysis. The mobile phase consisted of buffer A (0.1% FA in H_2_O) and buffer B (0.1% FA in acetonitrile), and the elution was performed with a mixture of buffer A and B in a ratio of 85:15 (v/v). The ESI voltage was 4.0 kV, the capillary temperature was 320°C, and SRM ion transition was selected based on an *m/z* value of 103.2.

## Results

### *ompW* mutation decreased the growth of *V. cholerae* in hypersaline culture conditions

**Figure 1:**
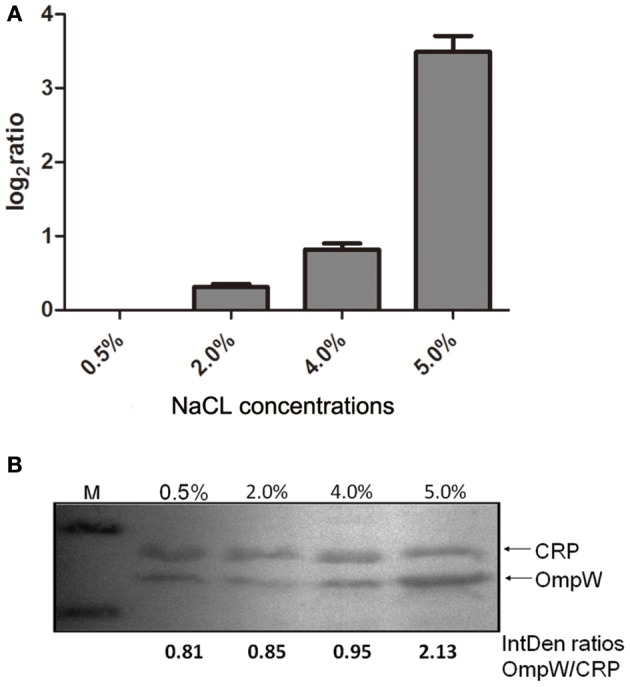
The level of *ompW* in *V. cholerae* El Tor strain C6706 exposed to salt stress. The *V. cholerae* wild-type strain C6706 was grown in M9 media containing different concentrations of NaCl (0.5, 2.0, 4.0, and 5.0%). **(A)** The level of *ompW* transcripts increased with increasing salt concentration, being up-regulated eight-fold at 5% NaCl. **(B)** Cell pellets were analyzed by SDS-PAGE and immunoblot assays using anti-OmpW and anti-CRP antibodies. The IntDen ratios of OmpW/CRP corresponding to the lanes of different NaCl concentrations were marked on the bottom of the figure.

**Figure 2:**
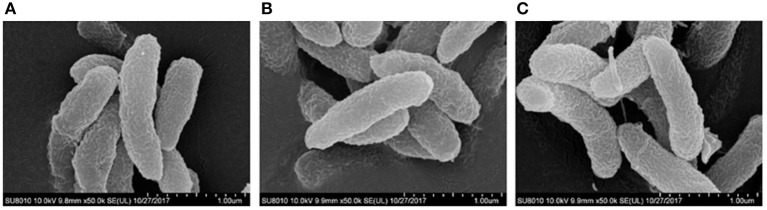
Scanning electron microscopy of *V. cholerae* strains C6706 **(A)**, C6706-Δ*ompW* **(B)**, and C6706-Δ*ompW/compW* **(C)**.

Considering the up-regulation of OmpW expression in response to high salinity in some bacteria (Wu et al., 2006), we measured the transcription and expression of the *ompW* gene of *V. cholerae* in media with different concentrations of NaCl. *V. cholerae* strain C6706 was cultivated in M9 media containing 0.5, 2, 4, or 5% NaCl. mRNA transcription analysis showed that the *ompW* gene was up-regulated after 1 h under high salt stress. In these M9 media, the transcription of *ompW* increased with increasing salt concentrations and was up-regulated eight-fold at 5% NaCl (Figure 1). OmpW expression in the M9 media with different concentrations of NaCl was further estimated with western blotting. Increased OmpW expression was observed at the high salt concentrations (Figure 1), and the IntDen ratios of the OmpW/CRP hybridization bands from the samples grown in M9 media containing 0.5, 2, 4, and 5% NaCl were 0. 37, 0.47, 0.55, and 1.91, respectively, showing that *ompW* may be a salt-sensitive gene. The changes in OmpW expression in the M9 media containing different NaCl concentrations were consistent with the *ompW* transcript levels. The SEM pictures showed that the shape of C6706, the *ompW* mutant C6706-Δ*ompW*, and its complementary strain C6706-Δ*ompW/compW* were identical (Figure 2).

**Figure 3:**
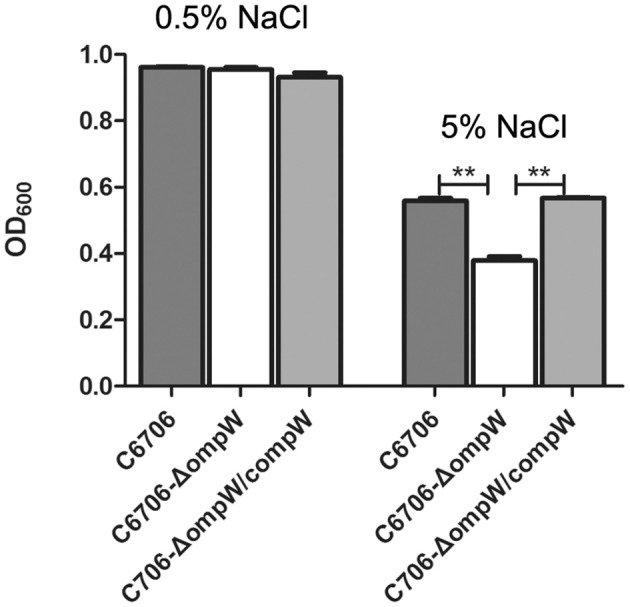
Growth of the *V. cholerae* wild-type strain C6706 (dark gray), C6706-Δ*ompW* (white), and C6706-Δ*ompW/compW* (gray) in M9 media containing 0.5% **(A)** and 5% NaCl **(B)**. The OD_600_ values were measured after 18 h of growth at 37°C and 200 rpm shaking. ^**^, Significant differences between the sample groups, two-tailed *t*-test (*P* < 0.005).

To measure the growth of the wild-type and *ompW* mutant strains in the hypersaline media, we cultivated C6706, the *ompW* mutant C6706-Δ*ompW*, and its complementary strain C6706-Δ*ompW/compW* in M9 media containing different concentrations of NaCl (0.5 and 5%) at 37°C. No significant differences were found in the growth of these three strains in M9 media containing 0.5% NaCl (Figure 3); however, at 5% NaCl, C6706-Δ*ompW*, compared with C6706, showed a slight but statistically significant decrease in growth, and the wild-type growth was restored when *ompW* gene was complemented in the C6706-Δ*ompW* cells (strain C6706-Δ*ompW/compW*) (Figure 3). We also surveyed the bacterial count and OD_600_ values continuously during 23 h of culturing. In the M9 supplemented with 0.5% NaCl, OD_600_ values of cultures with C6706, C6706-Δ*ompW*, and C6706-Δ*ompW/compW* cells were identical, whereas in the M9 culture with 5% NaCl, C6706-Δ*ompW* grew slower than wild-type C6706 and the *ompW* complementary strain C6706-Δ*ompW/compW* (Figures S1, S2). These results suggested that deletion of the *ompW* gene reduced the ability of the *V. cholerae* strain to grow under the studied salt stress conditions and that complementation of the *ompW* gene could restore the growth of the *ompW* mutant.

### *L*-carnitine, betaine, and *L*-lysine promoted the growth of *V. cholerae* in hypersaline media

**Figure 4:**
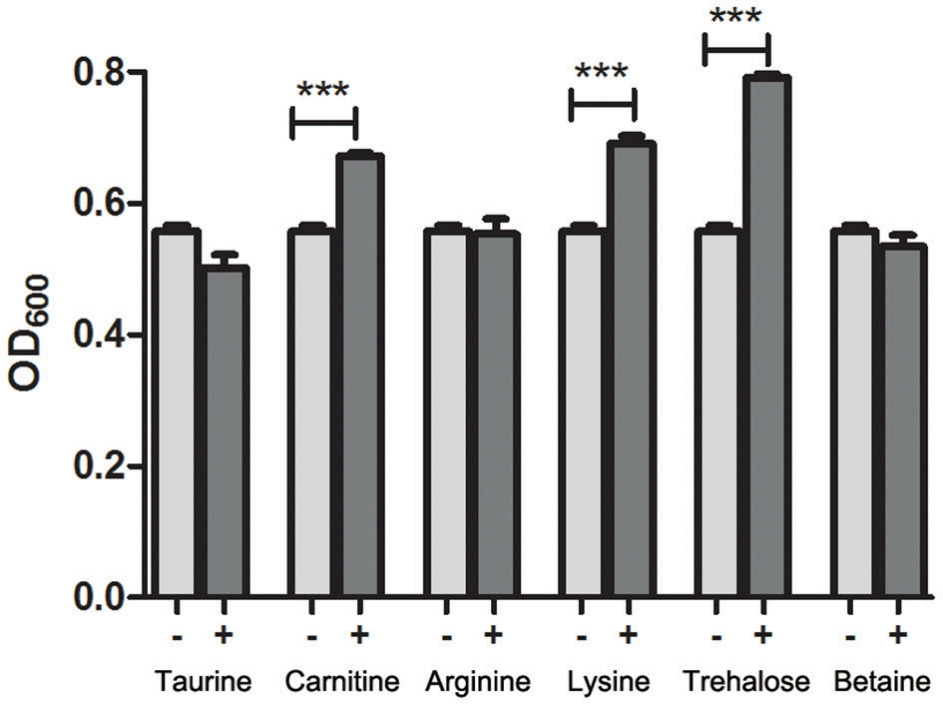
Growth of *V. cholerae* strain C6706 in M9 media containing 5% NaCl in the presence of various osmoprotectants. One of six osmoprotectants were each added to separate cultures, and the OD_600_ values of the cultures were measured after 18 h of growth at 37°C and 200 rpm shaking. “−” indicates no added osmoprotectant, “+” indicates the addition of osmoprotectant. ^***^, Significant differences between the sample groups, two-tailed *t*-test (*P* < 0.001).

To identify the compatible solutes that can promote the growth of *V. cholerae* in the media with high concentrations of NaCl, six candidate osmoprotectants, including *L*-carnitine, betaine, *L*-arginine, *L*-taurine, *L*-trehalose, and *L*-lysine, were added to the culture medium of strain C6706, and the growth of the cells was surveyed. The results showed that after 18 h of growth in the M9 medium containing 0.5% NaCl, no significant differences in OD_600_ values were observed in the strains cultivated in the presence or absence of all six osmoprotectants (Figure S3), whereas in the M9 media containing 5% NaCl, mean OD_600_ values in *V. cholerae* cultures significantly increased when the cells were grown in the presence of *L*-carnitine, betaine, or *L*-lysine. No growth differences were found in the media containing *L*-arginine, *L*-taurine, or *L*-trehalose (Figure 4), suggesting that *L*-carnitine, betaine, and *L*-lysine may act as osmoprotectants and play roles in the salt stress resistance of *V. cholerae*.

### OmpW conferred salt tolerance by transporting carnitine in *V. cholerae*

**Figure 5:**
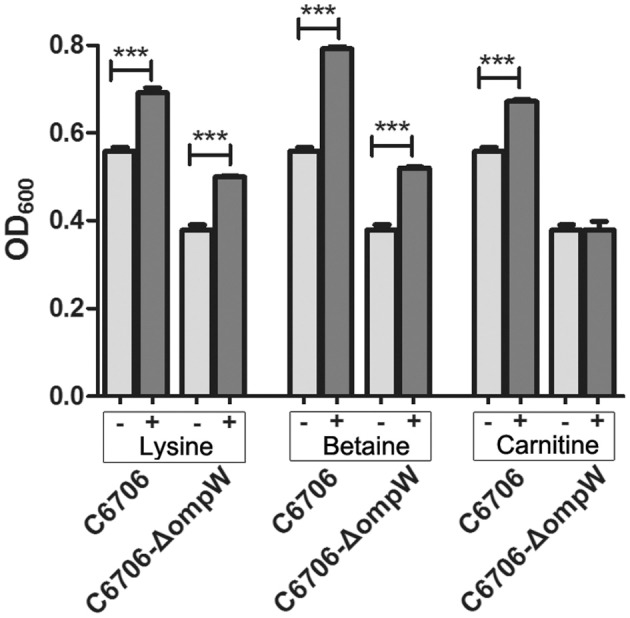
Growth of *V. cholerae* strain C6706 in M9 media with 5% NaCl in the presence of *L*-carnitine, betaine, and *L*-lysine. One of three osmoprotectants were each added to separate cultures, and the OD_600_ values of the cultures were measured after 18 h of growth at 37°C and 200 rpm shaking. “−” indicates no added osmoprotectant, “+” indicates the addition of osmoprotectant. ^***^, Significant differences between the sample groups, two-tailed *t*-test (*P* < 0.001).

OmpW transports small hydrophobic molecules. Here, we first screened the three osmoprotectants (*L*-carnitine, betaine, and *L*-lysine) that behaved differently in the above assays and may be substrates of OmpW in *V. cholerae*. When betaine and *L*-lysine were added to the M9 media at a concentration of 5 mM, the growth rates of the *V. cholerae* strains C6706 and C6706-Δ*ompW* were indistinguishable. However, following the addition of 5 mM *L*-carnitine, the growth rate of C6706-Δ*ompW* was significantly defective when compared to that of wild-type C6706 (Figure 5 and Figure S4), suggesting that *L*-carnitine is transported through OmpW.

**Figure 6:**
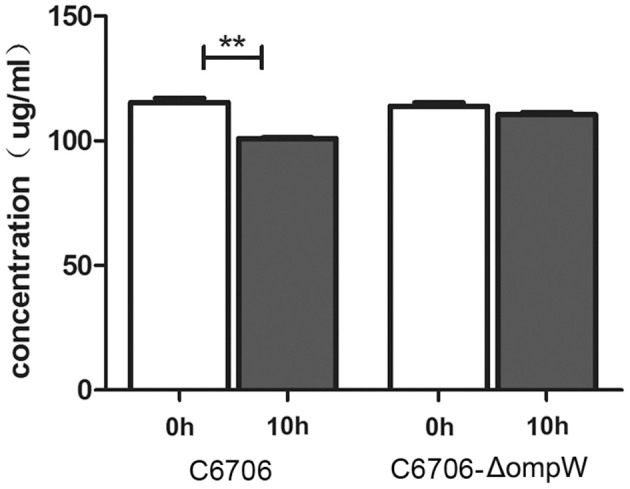
Quantitative analysis of carnitine by LC-MRM-MS. Carnitine concentrations in the supernatant of C6706 and C6706-Δ*ompW* cultures were measured at two time points (0 and 10 h). ^**^, Significant differences between the sample groups, two-tailed *t*-test (*P* < 0.005).

Further, the consumption of *L*-carnitine in the culture supernatant of *V. cholerae* strains was directly quantified by using LC-MRM-MS. A typical chromatogram of *L*-carnitine and the chromatograms of samples were shown in Figure S5, and the retention time of *L*-carnitine was identified at 11.75 min. The quantitative analysis of *L*-carnitine was thus achieved by the LC-MRM-MS method. The concentrations of *L*-carnitine in the supernatant of the culture media of strains C6706 and C6706-Δ*ompW* were quantified. Figure 6 shows that the concentrations of *L*-carnitine in C6706-Δ*ompW* cultures were indistinguishable at two time points (0 and 10 h). However, the concentration of *L*-carnitine in the C6706 culture was lower at 10 h than at 0 h (*p* < 0.05), which showed that OmpW plays a role in the transportation of *L*-carnitine.

**Figure 7:**
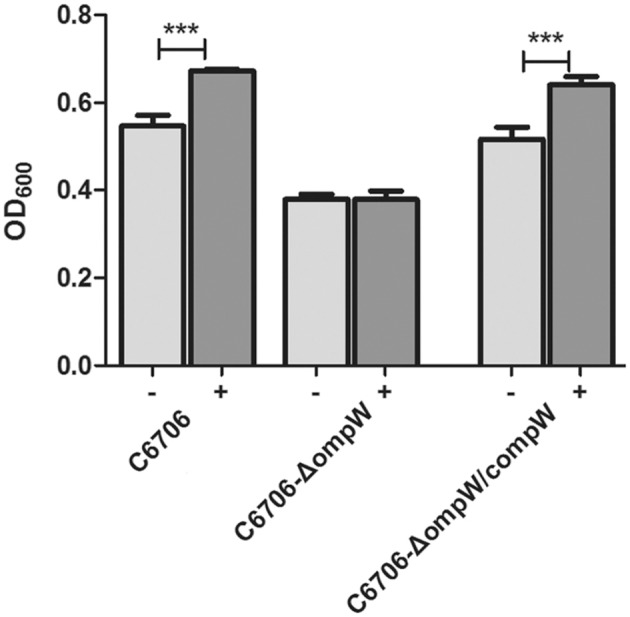
Growth of strains C6706, C6706-Δ*ompW*, and C6706-Δ*ompW/compW* in M9 media containing 5% NaCl in the presence of carnitine. The OD_600_ values of the cultures were measured after 18 h of growth at 37°C and 200 rpm shaking “−” indicates no added carnitine, “+” indicates the addition of carnitine. ^***^, Significant differences between the sample groups, two-tailed *t*-test (*P* < 0.001).

We then estimated the abilities of the wild-type and *ompW* mutant strains to grow under different NaCl concentrations and with/without *L*-carnitine. At 0.5% NaCl, the growth of the *V. cholerae* strains (C6706, C6706-Δ*ompW*, and C6706-Δ*ompW/compW*) showed no significant difference with or without *L*-carnitine (Figure S6). At the hypersaline NaCl concentration of 5%, the growth (OD_600_) of strains C6706 and C6706-Δ*ompW/compW* improved when 5 mM *L*-carnitine was supplied. However, no growth change was observed in the strain C6706-Δ*ompW*, even when *L*-carnitine was added to the M9 media (Figure 7). Considering these results, we deduced that OmpW plays a role in enhancing *V. cholerae* growth in hypersaline conditions.

## Discussion

As an important human intestinal pathogen and an inhabitant of estuarine water, *V. cholerae* relies on osmoadaptation to persist in high-salt environments. We have found that certain genes involved in Na^+^/K^+^ transport and glutamate biosynthesis and some sigma factors are sensitive to salt stress in *V. cholerae* (Fu et al., 2014); these genes responded in a variety of manners to a hypersaline environment. The outer membrane protein OmpW has been suggested to be involved in responses to various stresses in *E. coli* (Molloy et al., 2000) and *Borrelia burgdorferi* (Obonyo et al., 2002) and in salt tolerance in *P. damselae* (Wu et al., 2006), *V. alginolyticus* (Xu et al., 2004) and *V. parahaemolyticus* (Xu et al., 2005). In this study, we showed that OmpW enhanced *V. cholerae* growth in hypersaline conditions by transporting carnitine.

Some compatible solutes, including sugars, polyols, amino acids and amino acid derivatives, may help bacteria survive and respond to stress (Brown, 1976; Sleator and Hill, 2002; Roberts, 2004, 2005). For example, glycine betaine is the preferred compatible solute and can be utilized by many bacteria to respond to osmostress (Boch et al., 1994, 1996; von Blohn et al., 1997). Although *V. cholerae* does not synthesize glycine betaine, it can accumulate glycine betaine generated by other bacteria in the microbial community under high-salt conditions via OpuD and PutP (Kapfhammer et al., 2005). *V. cholerae* may also synthesize ectoine and transport proline to enhance its salt tolerance (Pflughoeft et al., 2003; Kapfhammer et al., 2005). In this study, we found that lysine, betaine, and carnitine are compatible solutes that improve the growth of *V. cholerae* under hypersaline condition, expanding the pool of compatible solutes that can be utilized by *V. cholerae* for its osmoadaptation.

OmpW of *V. cholerae* is a 22 kDa outer membrane protein (Manning et al., 1985; Jalajakumari and Manning, 1990), is conserved, and has been used as a target gene for the detection and identification of *V. cholerae* (Nandi et al., 2000). We also found that OmpW acts as the receptor for *V. cholerae* typing phage VP5 (Xu et al., 2014). In some species of bacteria, high NaCl concentrations induced ompW expression (Xu et al., 2004, 2005). OmpW belongs to the OmpW/AlkL family and has an 8-stranded β-barrel that forms a long and narrow channel be involved in the transport of small molecules across the bacterial outer membrane. (van Beilen et al., 2001; Hong et al., 2006). OmpW is involved in the transport of iron in *Shewanella oneidensis* (Thompson et al., 2002) and may transport the charged quaternary ammonium compound methyl viologen to outside of the cell in *Salmonella* typhimurium (Gil et al., 2007) and may indicated to participate with small multidrug resistance protein member EmrE to expel quaternary cationic compounds(Beketskaia et al., 2014). In our study, OmpW was associated with transport the compatible solute carnitine and enhanced the growth of *V. cholerae* under hypersaline conditions. Carnitine is often present and sometimes abundant in soil and natural waters. In the environments, carnitine was the most abundant quaternary ammonium compound (0.49 mM). The carnitine levels in soil and water may vary depending on the bacterial flora at the site and whether the bacteria inhabiting those environments are capable of carnitine metabolism (Warren, 2013). Though the deletion of *ompW* did not completely obstruct the growth of the mutant strain in hypersaline M9 media, it reduced mutant strain growth; however, wild-type growth could be restored in the mutant strain by complementing the *ompW* gene. In fact, there are multiple pathways of hypersaline tolerance in *Vibrio* (van Beilen et al., 2001; Hong et al., 2006), and many factors contribute to salt tolerance in *V. cholerae*. OmpW therefore played a role in enhancing *V. cholerae* growth in hypersaline conditions. It is reported that OmpW proteins can specifically bind molecules that are present in the extracellular environment such as LDAO and fumarate (Hong et al., 2006; Huang et al., 2006; Xiao et al., 2016). The hydrophobic tail of the LDAO molecule is close to the hydrophobic residues and the polar head group is located at the end of the barrel (Hong et al., 2006). By the docking and the algorithm, fumarate is shown to bind to aside pocket of OmpW and differ from that of LDAO (Huang et al., 2006; Xiao et al., 2016). We used the shake-flask method to identify the carnitine octanol/water partition coefficients (Engelmann et al., 2007). The result showed that the Kow of carnitine was less than 1 that demonstrated carnitine was a hydrophilic substance (data not shown). Like LDAO and fumarate, the carnitine is also small molecules dissolved in water which also contains hydrophobic groups and hydrophilic groups. Therefore, we hypothesized that the pattern carnitine transfer into cells through OmpW maybe likely to be similar to LDAO. In view of the fact that carnitine was defective in transportation in the *ompW* deletion mutant compared to the wild type strain, we deduce that carnitine may bind directly to OmpW as well, but their virtual structural interaction is needed to be confirmed experimentally in the future.

Our findings demonstrate that one of the roles of OmpW is to increase salt tolerance in *V. cholerae* by importing carnitine from the environment. Our study also expands the understanding of the biological role of OmpW in *V. cholerae*.

## Author contributions

BK conceived the idea, directed the work, designed the experiments, and revised the manuscript; XF performed the experiments, analyzed the data, and wrote the manuscript; JZ and JL contributed to the plasmid construction; TL and MZ provided technical support.

### Conflict of interest statement

The authors declare that the research was conducted in the absence of any commercial or financial relationships that could be construed as a potential conflict of interest.

## Supplementary material

Figure S1 Growth curves for *V. cholerae* strains C6706, C6706-Δ*ompW*, and C6706-Δ*ompW/compW* grown in M9 media containing 0.5% **(A)** and 5% NaCl **(B)**.

Figure S2 The bacteria count for *V. cholerae* strains C6706, C6706-Δ*ompW*, and C6706-Δ*ompW/compW* grown in M9 media containing 5% NaCl.

Figure S3 Growth of *V. cholerae* strain C6706 in M9 media containing 0.5% in the presence of various osmoprotectants. One of six osmoprotectants were each added to separate cultures, and the OD_600_ values of the cultures were measured after 18 h of growth at 37°C and 200 rpm shaking. “−” indicates no added osmoprotectant, “+” indicates the addition of osmoprotectant.

Figure S4 Growth of *V. cholerae* strains C6706 and C6706-Δ*ompW* in M9 media with 5% NaCl in the presence of *L*-lysine **(A)**, betaine **(B)**, and *L*-carnitine **(C)**. One of three osmoprotectants were each added to separate cultures.

Figure S5 The typical chromatogram of *L*-carnitine and the chromatograms of samples. **(A)**: A typical chromatogram; **(B)**: C6706 (0 h); **(C)**: C6706-Δ*ompW* (0 h); **(D)**: C6706 (10 h); **(E)**: C6706-Δ*ompW* (10 h).

Figure S6 Growth of strains C6706, C6706-Δ*ompW*, and C6706-Δ*ompW/compW* in M9 media containing 0.5% in the presence of carnitine. The OD_600_ values of the cultures were measured after 18 h of growth at 37°C and 200 rpm shaking “−” indicates no added carnitine, “+” indicates the addition of carnitine.
