# Supplementary figures and images for: The Outer Membrane Protein OmpW Enhanced V. cholerae Growth in Hypersaline Conditions by Transporting Carnitine
Source: Front Microbiol. 2018 Jan 22;8:2703. doi: 10.3389/fmicb.2017.02703 (PMC5786537; doi:10.3389/fmicb.2017.02703)

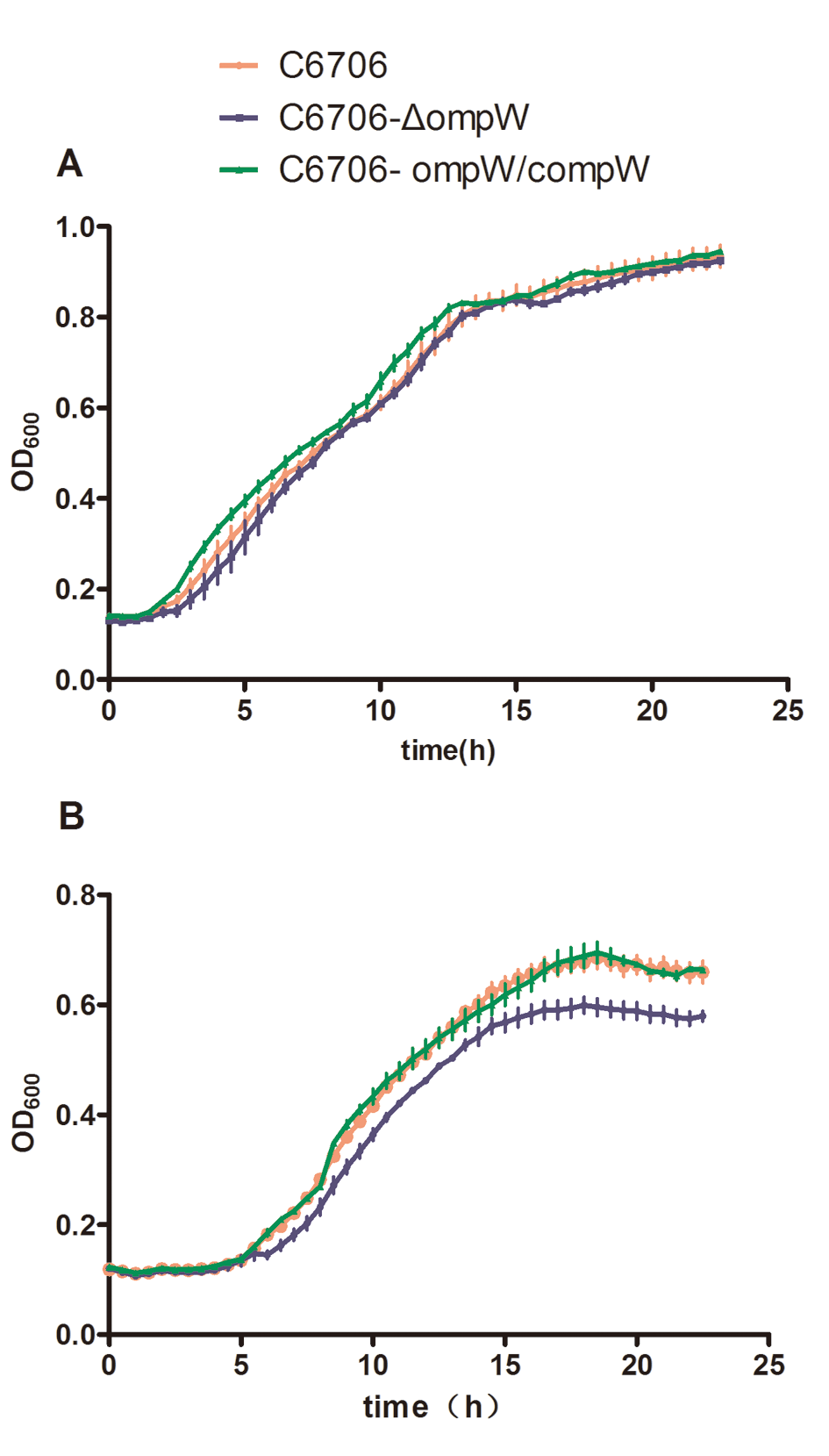

Supplement: Figure S1 — Growth curves for V. cholerae strains C6706, C6706-ΔompW, and C6706-ΔompW/compW grown in M9 media containing 0.5% (A) and 5% NaCl (B). [file Image1.TIF]

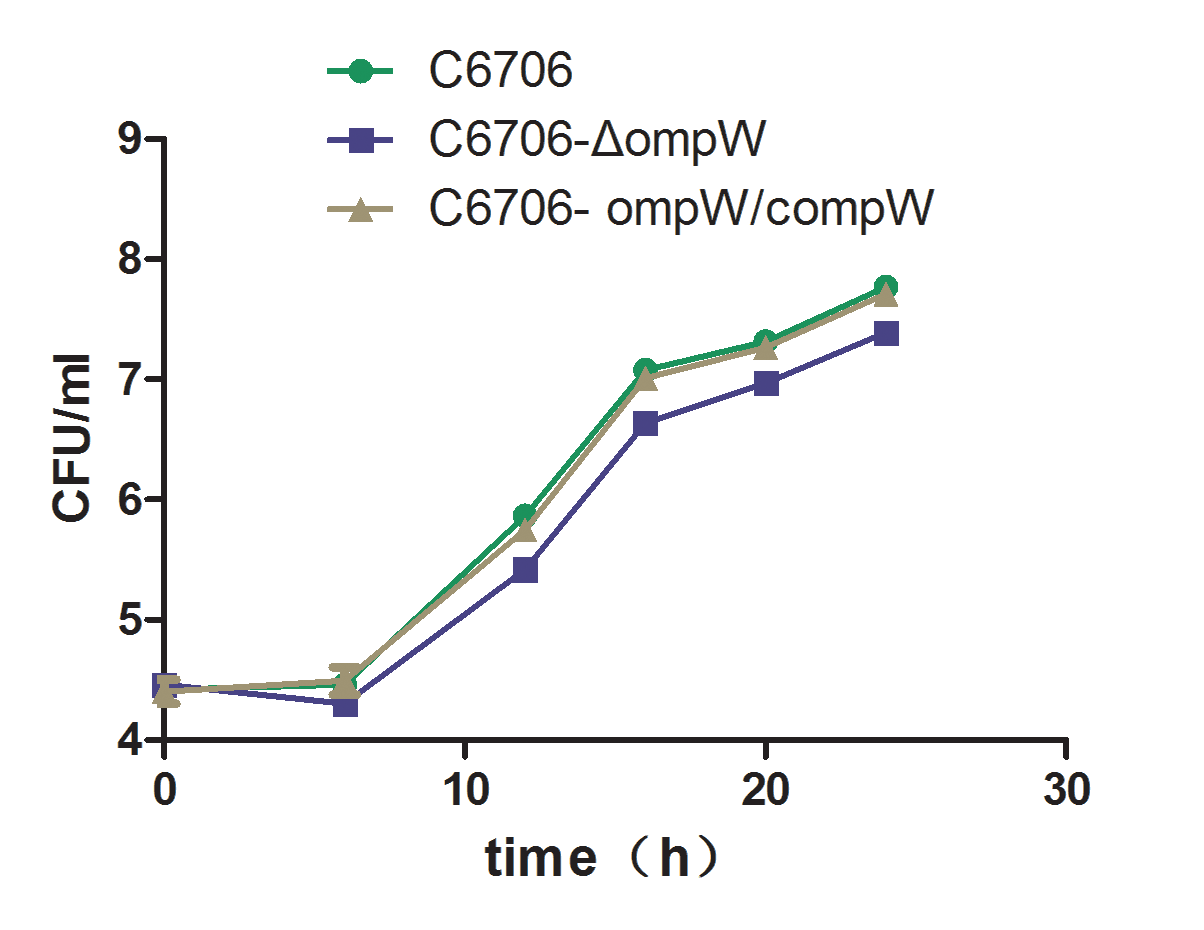

Supplement: Figure S2 — The bacteria count for V. cholerae strains C6706, C6706-ΔompW, and C6706-ΔompW/compW grown in M9 media containing 5% NaCl. [file Image2.TIF]

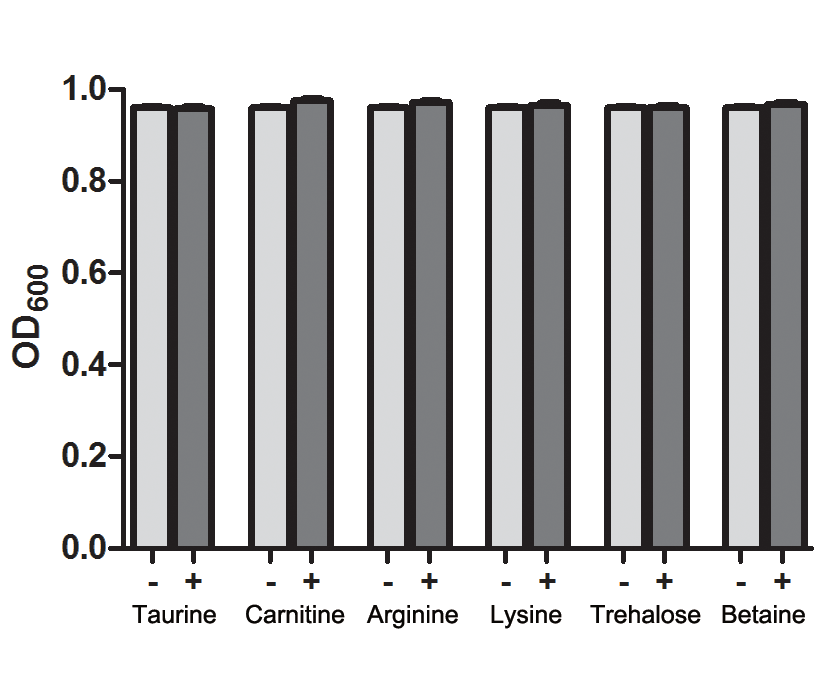

Supplement: Figure S3 — Growth of V. cholerae strain C6706 in M9 media containing 0.5% in the presence of various osmoprotectants. One of six osmoprotectants were each added to separate cultures, and the OD600 values of the cultures were measured after 18 h of growth at 37°C and 200 rpm shaking. “−” indicates no added osmoprotectant, “+” indicates the addition of osmoprotectant. [file Image3.TIF]

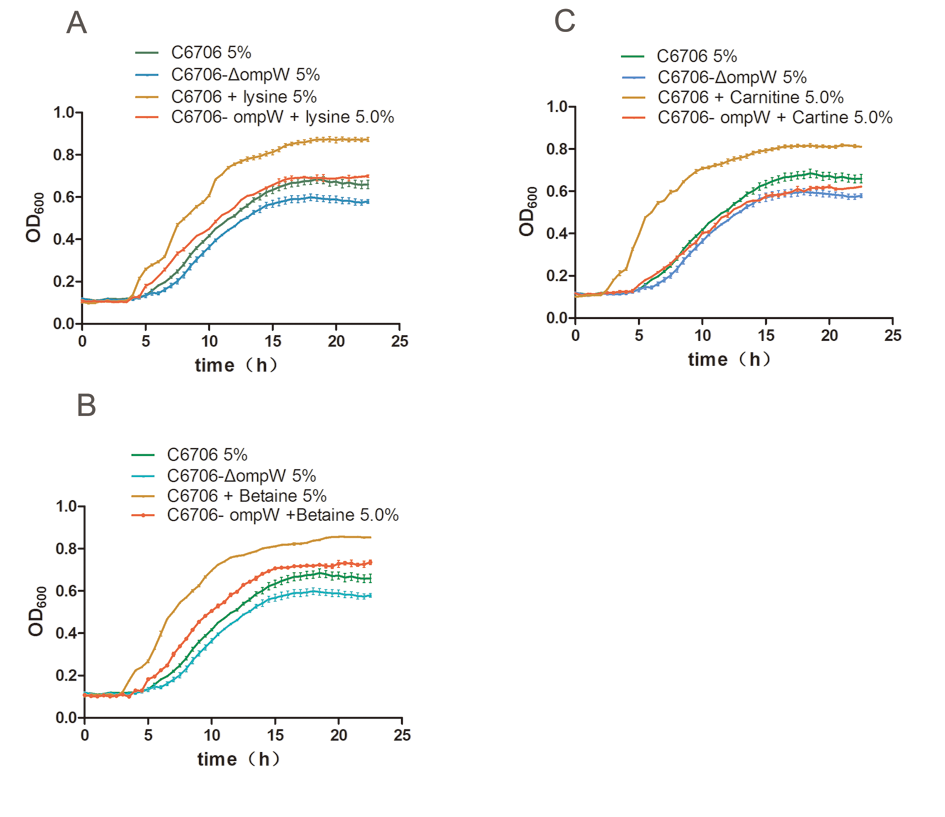

Supplement: Figure S4 — Growth of V. cholerae strains C6706 and C6706-ΔompW in M9 media with 5% NaCl in the presence of L-lysine (A), betaine (B), and L-carnitine (C). One of three osmoprotectants were each added to separate cultures. [file Image4.TIF]

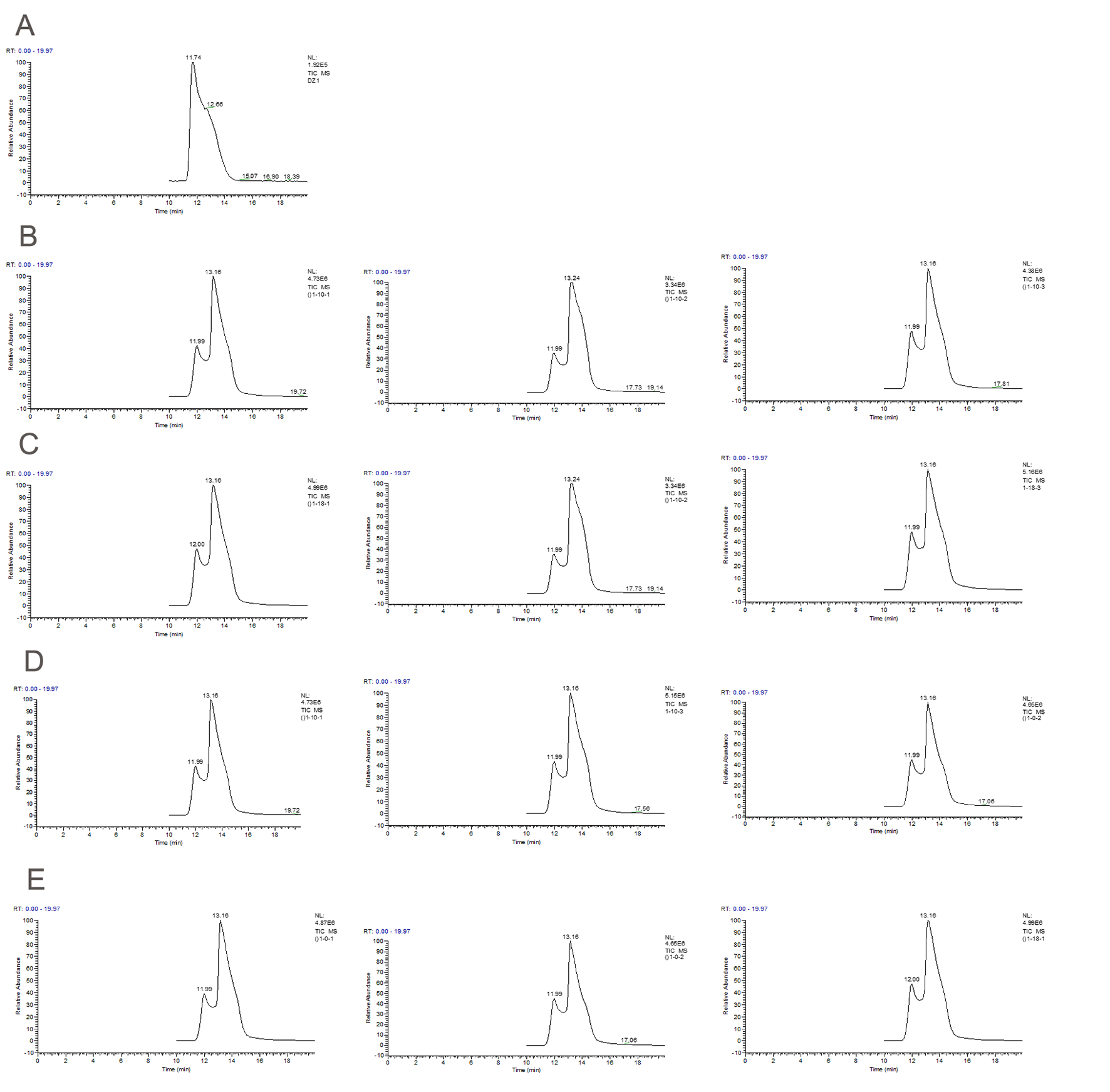

Supplement: Figure S5 — The typical chromatogram of L-carnitine and the chromatograms of samples. (A): A typical chromatogram; (B): C6706 (0 h); (C): C6706-ΔompW (0 h); (D): C6706 (10 h); (E): C6706-ΔompW (10 h). [file Image5.TIF]

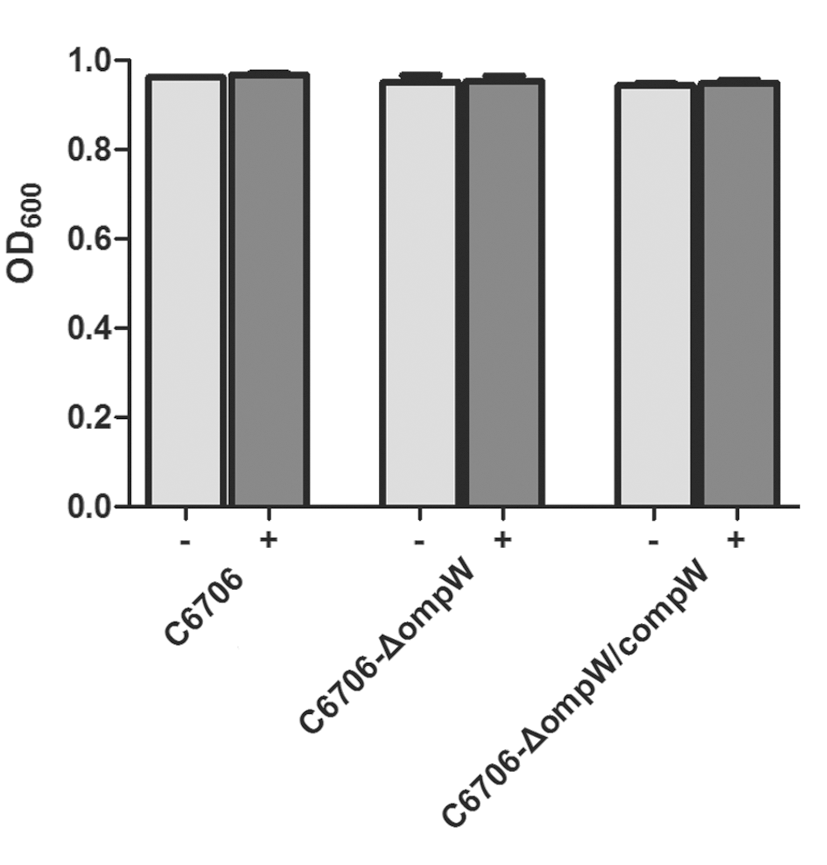

Supplement: Figure S6 — Growth of strains C6706, C6706-ΔompW, and C6706-ΔompW/compW in M9 media containing 0.5% in the presence of carnitine. The OD600 values of the cultures were measured after 18 h of growth at 37°C and 200 rpm shaking “−” indicates no added carnitine, “+” indicates the addition of carnitine. [file Image6.TIF]
